# Headspace Solid-Phase Microextraction Coupled to Comprehensive Two-Dimensional Gas Chromatography Time-of-Flight Mass Spectrometry for the Determination of Short-Chain Chlorinated Paraffins in Water Samples

**DOI:** 10.1155/2018/2768547

**Published:** 2018-10-01

**Authors:** Nan Zhan, Feng Guo, Shuai Zhu, Zhu Rao

**Affiliations:** The Key Laboratory of Eco-Geochemistry, Ministry of Nature Resources and National Research Center for Geoanalysis, Beijing 100037, China

## Abstract

Short-chain chlorinated paraffins (SCCPs) are a new type of persistent organic pollutants. In this work, a simple and effective method involving headspace solid-phase microextraction (HS-SPME) and comprehensive two-dimensional gas chromatography time-of-flight mass spectrometry (GC × GC-TOF-MS) was developed and optimized for the determination of trace SCCPs in water samples. The key parameters related to extraction and separation efficiency were systematically optimized. The SCCP congener groups were best resolved using an Rxi-5Sil MS (30 m × 0.25 mm × 0.25 *µ*m) column followed by an Rxi-17Sil MS (1.0 m × 0.15 mm × 0.15 *µ*m) column; the optimum extraction conditions were achieved with a 100 *µ*m polydimethylsiloxane SPME fiber, when a 10 mL water sample added with 3.6 g sodium chloride was incubated for 15 min at 90°C and then extracted during 60 min at 90°C and desorption at 260°C for 2 min. The proposed method showed good linearity in the concentration range of 0.2–20.0 *µ*g/L with the determination coefficient greater than 0.995. The detection and quantification limits ranged from 0.06 to 0.13 *µ*g/L and 0.18 to 0.40 *µ*g/L, respectively, which are sufficient to meet the regulatory detection limits as set by most environmental regulations. The accuracy and precision of the method was also good, where the recoveries ranged from 82.5 to 95.4%, and intra- and interday precision was within 7.2% and 14.5%, respectively. The optimized method has been applied to the determination of SCCPs in ten freshwater samples of three different types.

## 1. Introduction

Short-chain chlorinated paraffins (SCCPs) are complex mixtures of polychlorinated *n*-alkanes with carbon chain lengths from 10 to 13 carbon atoms and with a chlorination degree in the range 30–70%. Because of their excellent physicochemical properties, SCCPs have been widely used in metalworking applications and polyvinyl chloride processing and used as plasticizers or flame retardants in paints, adhesives, sealants, leather fat liquor, lubricants, plastics, rubber textiles, and polymeric materials since the 1930s [1–3]. However, in the last two decades, SCCPs have been found to be toxic towards aquatic organisms and persistent in the environment and have high potential for bioaccumulation and long-distance transport at relatively low concentrations. Since then, their production and use have been gradually restricted and included in some environmental laws and regulations. In Japan, SCCPs are listed in Pollutant Release and Transfer Register Law and are no longer manufactured [1, 2]; in Canada, SCCPs have been included in the “first Priority Substances List” under the Canadian Environmental Protection Act [1, 2, 4]; and in Europe, SCCPs are no longer permitted for sale or manufacture and have been listed as hazardous priority substances in the European Water Framework Directive [1, 2, 5]. Not long ago, SCCPs were officially included in the Annex A of Stockholm Convention [6] and Annex III of Rotterdam Convention [7], indicating that their production, use, and emission will soon be limited worldwide.

Among various environmental matrices, water is the most important environmental sink for SCCPs by either direct emission or from sewage treatment systems [1, 8]. In most cases, the presence of SCCPs in freshwater or seawater is reported at low concentrations (*µ*g/L level or below) [1, 2]. Hence, suitable sample preparation methods and reliable instrumental analysis methods are much required for the determination of SCCPs in water environment.

Over recent years, liquid-liquid extraction (LLE) [8, 9] and solid-phase extraction (SPE) [10–12] are widely used sample preparation techniques for the preconcentration of SCCPs from water samples. However, LLE [8, 9] is time-consuming and requires large quantities of organic solvent; SPE [10–12] is susceptible to high baseline blanks, sorbent bed blocking, and poor reproducibility problems; and both methods cannot avoid the loss of the sample. The development of solid-phase microextraction (SPME) technique overcame the traditional sample preparation procedure, integrating extraction, concentration, and sample introduction in a single step, thus significantly saving time and labor [13]. Moreover, SPME only requires a few milliliters of water samples and does not require organic solvent, which is an added advantage. With the automation technology, the automated online SPME has realized in situ sample preparation with high efficiency and has been widely used for the analysis of volatile and semivolatile organic compounds in water samples [14–17]. However, the application of the SPME technique for extracting SCCPs from water samples is still limited and scarcely reported [10, 14, 17]. Gandolfi et al. [17] compared SPME in the direct immersion mode (DI-SPME) and headspace mode (HS-SPME) for the study of SCCPs in water samples and found that HS-SPME is preferable than DI-SPME, as it could reduce the extraction time, extend the service life of the extraction fiber, and prevent the matrix effect as well as keep the instrument system (e.g., GC column, MS) cleaner. Therefore, the HS-SPME method was selected in this study based on its numerous advantages.

The instrumental methods for the determination of SCCPs, just like many halogenated compounds, are mainly based on GC with different detectors, especially with low-resolution MS. Since SCCPs have thousands of isomers, enantiomers, and diastereomers in the mixtures, a single capillary GC column is insufficient to resolve all the congeners as individual peaks, always resulting a characterized “hump” peak in the chromatogram as unresolved complex mixture [18–23]. The state of art for SCCP analysis is the use of comprehensive two-dimensional gas chromatography (GC × GC) that significantly improves the peak capacity by using two GC columns with different retention mechanisms, allowing the SCCP congeners to be separated into ordered structures according to the carbon chain length and number of chlorine atoms on the chromatogram [24–26]. Moreover, with improved chromatographic separation, SCCPs can also be better separated from the interference compounds such as polychlorinated biphenyls and from complex matrices at the same time [25, 26]. Xia et al. demonstrated the feasibility of GC × GC for quickly screening and separating SCCPs in a fish sample, not only improving the detection and separation of SCCPs but also enabling separation of the SCCP congeners from other coeluting halogenated organic compounds [25].

Currently, time-of-flight mass spectrometry (TOF-MS) is the most commonly used mass spectroscopy technique connected to GC × GC, whose fast acquisition rates allow proper reconstruction of 2D chromatogram by producing hundreds of spectra per second. The electron capture negative ionization mode (ECNI) has been extensively used for the quantification of SCCPs [18–23] because of its capability to differentiate the structural isomers of SCCPs by less fragmentation at low ionization energies, thus enhancing the selectivity of the method. However, in the ECNI mode, the response factors depend heavily on the number of chlorine atoms and their position in the carbon chain [1–3, 27], making the detection of the lower chlorinated SCCPs congeners (Cl < 5) difficult, resulting in quantitative errors [1, 3]. Contrary to ECNI, electron impact (EI) ionization is independent of the chlorine content and carbon chain length and thus can overcome the chlorination discrimination flaw; therefore, EI-MS can also detect lower chlorinated SCCPs (Cl < 5). This is important because lower chlorine components accounted for 10–62% mass of the total content in the environmental samples and are not negligible [28, 29]. The EI mode is known to lead to extensive fragmentation, making the mass spectra difficult to interpret. In other words, EI-MS cannot identify congener and homologue patterns; therefore, it is usually used for the detection of total concentration of SCCPs in the sample [1–3, 20].

At present, to the best of our knowledge, online HS-SPME coupled to GC × GC-TOF-MS for the determination of SCCPs in water samples has not been reported. Therefore, the aim of this study was to develop a HS-SPME GC × GC-TOF-MS analytical method for the determination of SCCPs in water samples. The key parameters related to extraction and separation efficiency were first carefully optimized. Then, the performance of the developed method was validated with respect to linearity, limit of detection (LOD), limit of quantification (LOQ), accuracy, and precision. Finally, the proposed method was applied to ten freshwater samples of three different types.

## 2. Materials and Methods

### 2.1. Chemicals and Materials

Commercial standards of SCCPs (C_10–13_, 55.5% chlorination; 100 *µ*g/mL in cyclohexane), medium-chain chlorinated paraffins (MCCPs, C_14–17_, 52% chlorination; 100 *µ*g/mL in cyclohexane), and long-chain chlorinated paraffins (LCCPs, C_18–20_, 49% chlorination; 100 *µ*g/mL in cyclohexane) were purchased from Dr. Ehrenstorfer GmbH (Augsburg, Germany). ^13^C_6_-hexachlorobenzene (100 *µ*g/mL in nonane) used as the internal standard was purchased from Cambridge Isotope Laboratories (Andover, USA). Acetone and cyclohexane of pesticide residue analysis grade and sodium chloride (NaCl) of analytical grade were purchased from J&K Scientific Limit (Beijing, China). Before use, NaCl was purified in a furnace oven at 450°C for 5 h. Ultrapure water was obtained from a Milli-Q purification system (Millipore, Bedford MA, USA). The stock solutions of SCCPs (C_10–13_, 55.5% chlorination) were prepared in two different solvents, acetone and cyclohexane, at 10 *µ*g/mL and stored at 4°C until use.

Three commercially available SPME fibers, 65 *µ*m polydimethylsiloxane/divinylbenzene (PDMS/DVB), 85 *µ*m polyacrylate (PA), and a 100 *µ*m polydimethylsiloxane (PDMS), were purchased from Sepelco (Sigma Aldrich, USA). All these fibers were preconditioned in a GC injection port according to the manufacturer's recommendations prior to use. 20 mL headspace SPME vials, polytetrafluoroethylene septum, and crimp caps were obtained from GERSTEL (Mülheim, GER).

### 2.2. Water Samples and Sample Preparation

Ten water samples including three types of freshwater were collected at different sites in Beijing (China). Two groundwater samples were collected from a tap of a resident and a tap of our laboratory. Four lake water samples and four river water samples were collected from Bayi lake and Kunyu river at different sampling points on October 2017. All the samples were collected in 500 mL amber glass bottles and preserved at 4°C. Before analysis, all water samples were filtered through a 0.45 *µ*m membrane filter (J&K, Beijing, China) to remove particulate matter.

### 2.3. HS-SPME Procedure

The HS-SPME procedure was performed in a 20 mL SPME vial containing a 10 mL water sample with 20 ng internal standard. The initial experimental conditions were based on the report by Gandolfi et al. [17] and are as follows: water sample was first stabilized in a thermal-static incubator for 10 min at 90°C; then, the SPME fiber was exposed in the headspace above the sample for 80 min at 90°C, followed by desorption on the GC injection port at 260°C for 2 min. After optimizing several key factors of the SPME step, the optimum extraction conditions were obtained: 10 mL water sample with the addition of 3.6 g NaCl was incubated at 90°C for 15 min and then extracted for 60 min at the same temperature, and finally, the fiber was desorbed in the GC injector at 260°C for 2 min. After desorption, fibers were reconditioned for 30 min at 260°C for removing the memory effect.

### 2.4. GC × GC-TOF-MS Conditions

The GC × GC-TOF-MS system was built from an Agilent 7890B GC (Agilent Technologies, Santa Clara, CA, USA) coupled to a Pegasus 4D TOF-MS (LECO, St. Joseph, MI, USA). The system was equipped with a multipurpose autosampler (GERSTEL, Mülheim, GER), including an SPME conditioning incubator and a temperature-controllable sample tray for 20 mL SPME vials. Liquid nitrogen, automatically filled from a Dewar using a liquid meter, was used to cool the nitrogen gas for cold pulses. Instrument control and data processing were performed by ChromaTOF software, version V4.51 (Leco, St. Joseph, MI, USA).

Based on the previous reports [24–26] and our preliminary experiment, a combination of nonpolar and midpolar column was found to be suitable for the analysis of SCCPs in the GC × GC system. Therefore, in this study, the first-dimension and second-dimension columns were an Rxi-5Sil MS column (5% phenyl + 95% methylpolysiloxane, Restek) and an Rxi-17Sil MS column ((50%-diphenyl)-dimethyl polysiloxane, Restek, 1.0 m × 0.15 mm × 0.15 *µ*m), respectively. The injection temperature was 260°C in the splitless mode, and helium (purity 99.999%) was used as the carrier gas. The optimum temperature program of the main oven was obtained from the experiments, comprising an initial temperature 100°C for 1 min, a ramp of 20°C/min to 150°C followed by a ramp of 3°C/min to 290°C and held for 1 min. The temperature of the secondary oven was programmed 5°C above the primary oven gradient. The modulator had a 15°C offset above the second oven. The TOF-MS was operated in the electron impact (EI) mode with ionization energy of 70 eV. Mass spectra were collected in the full-scan mode over the *m*/*z* range 50–500. Ion source and transfer line temperatures were set as 240 and 280°C, respectively. Other instrument parameters such as modulation period, gas flow rate, and MS acquisition rate were obtained from the results of optimized experiments, as described in Section 3.1.

### 2.5. Identification and Quantification

The identification of SCCPs in real samples was performed by comparing their elution area and retention times with that of standard solutions and by comparing their mass spectra with the reference mass spectra from the NIST (National Institute of Standards and Technology) library. A minimum similarity value of 750 was applied.

For quantification of SCCPs, the seven-point standard calibration curves were constructed at the 0.2, 0.5, 1, 2, 5, 10, and 20 ng/mL concentration levels by spiking the blank water samples with the appropriate amount of the stock solutions and 20 ng internal standard. Since EI-MS uses high energetic electrons to produce fragment ions and thus leads to extensive fragmentation, the molecular characteristic peaks are usually weak. Therefore, the quantification is based on the characteristic fragment ions of low mass-to-charge ratio values (*m*/*z*). Here, the fragment ion at *m*/*z* 91 and *m*/*z* 187 was selected as the quantitative ion for SCCPs and internal standard, respectively. The LODs and LOQs of the method were determined as three and ten times the SD of the average values of seven blank water samples, respectively. The accuracy of this method was tested by the recovery studies of blank water samples spiked with 1.0, 5.0, and 10.0 *µ*g/L SCCP standards in five replicates. The recoveries are calculated by comparing the peak area ratio obtained with a 100 *µ*m PDMS fiber in DI-SPME, which were the optimal conditions established by Gandolfi et al. [17]. The precision of the method was evaluated by intraday and interday reproducibility experiments by analyzing the spiked water sample (10.0 *µ*g/L) for five times on the same day and daily for five times over three different days.

## 3. Results and Discussion

### 3.1. Optimization of GC × GC-EI-TOF-MS

The GC × GC-TOF-MS conditions were optimized to achieve good separation of SCCP congeners, by analyzing a SCCP standard solution (C_10–13_, 55.5% chlorination in cyclohexane) at 10 *µ*g/mL in the splitless mode. Each experiment was performed in triplicate.

**Figure 1:**
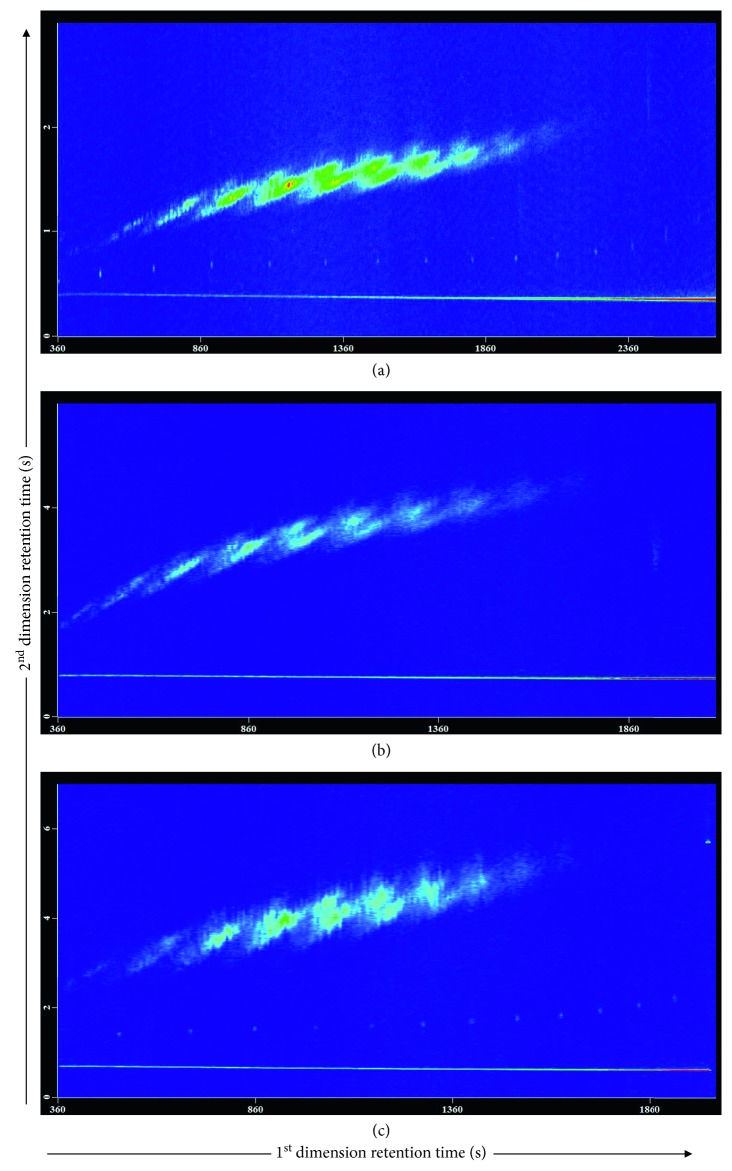
The effect of column length and film thickness of the primary column Rxi-5Sil MS on the separation performance of SCCP congeners. GC × GC-TOF-MS chromatograms of a mixed SCCP standard solution (C_10-13_, containing 55.5% chlorine), acquired using (a) 30 m × 0.25 mm × 0.25 *µ*m, (b) 15 m × 0.25 mm × 0.15 *µ*m, and (c) 30 m × 0.25 mm × 0.15 *µ*m Rxi-5Sil MS columns.

Since the column dimension could essentially affect the retention behavior of target compounds (i.e., SCCPs) on GC, the column length and film thickness of the primary column were first optimized. Three Rxi-5Sil MS columns were studied with a certain second column Rxi-17Sil MS (1.0 m × 0.15 mm × 0.15 *µ*m). Accordingly, the modulation period was adjusted to ensure all the SCCP congeners eluting in one modulation period. The results revealed that SCCPs can be resolved into an ordered structure on the 2D chromatogram by all three columns, where the SCCPs are arranged by boiling points on the *x*-axis and by polarity on the *y*-axis, far better than that obtained by 1D GC. And this typical “tile-structure” of SCCPs helps them to be identified quickly in the sample. As can be seen from Figure 1, a narrow-bore column (30 m long and 0.15 *µ*m film thickness) and a short narrow-bore column (15 m long and 0.15 *µ*m film thickness) decreased the retention of target compounds on GC and thus accelerated their elution; therefore, a relatively long modulation period (e.g., 6 s or 7 s) was required to avoid the wraparound effect. But using such a long modulation period, the separation efficiency achieved on the primary column decreased, and the peak shape and peak intensity deteriorated too (Figures 1 and 1). However, a regular size column (30 m long and 0.25 *µ*m film thickness) could separate SCCPs well in a quite short modulation period such as 3 s (Figure 1). In addition, regarding the anti-interference ability, a thin and/or short GC column is more susceptible to matrix interference than one of the regular dimension columns, especially for matrix-rich environmental samples such as wastewater sample, while a regular dimension column is more adaptable and robust for analyzing various water samples. Therefore, a 30 m long, 0.25 mm internal diameter, and 0.25 *µ*m film thickness Rxi-5Sil MS column was selected in this study.

**Figure 2:**
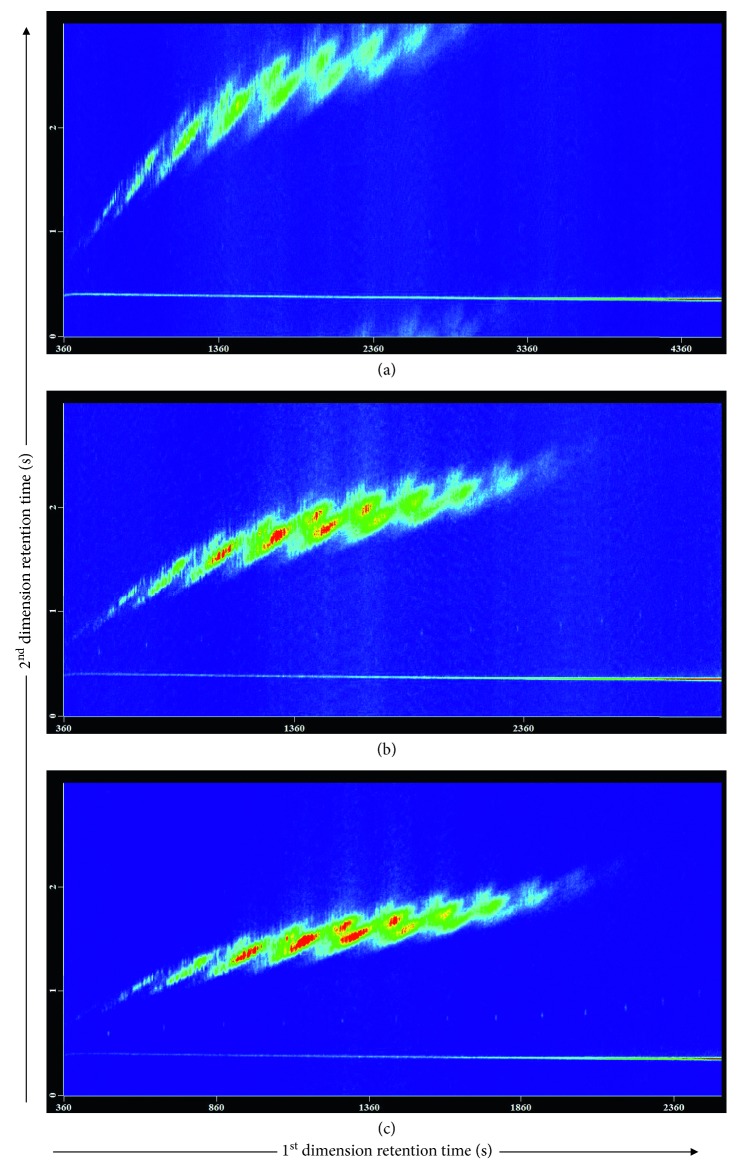
The effect of the heating rate on the separation performance of SCCP congeners. Temperature program of the primary oven: 100°C (1 min), at 20°C/min to 150°C, and then at *X*°C/min to 290°C (1 min). *X* = (a) 2°C/min, (b) 3°C/min, and (c) 4°C/min.

The GC oven temperature program was then optimized for the optimum extent of the SCCP congeners. In this experiment, heating rates from 2 to 4°C/min were evaluated in the main oven temperature programs. The initial temperature program used is as follows: held for 1 min at 100°C, increased to 150°C at 20°C/min, and finally ramped to 290°C at *X*°C/min (*X* = 2, 3, and 4) and held for 1 min. A heating rate of 2°C/min (Figure 2) provided the best separation of SCCP congeners but caused a “wraparound” for some high-boiling-point SCCPs. When the heating rate was increased to 3°C/min (Figure 2), all SCCP congeners eluted in one modulation period with good separation, together with providing a more efficient laboratory output in a relatively short run time (51.5 min) and reducing the liquid nitrogen consumption. At a higher heating rate such as 4°C/min, some neighboring groups of SCCPs overlapped and coeluted, leading to worse separation (Figure 2). Therefore, 3°C/min was selected as the optimum heating rate. For this, the initial temperature of the first oven was programmed at 100°C and held for 1 min, then ramped to 150°C at 20°C/min, and further increased to 290°C at 3°C/min and held for 1 min.

**Figure 3:**
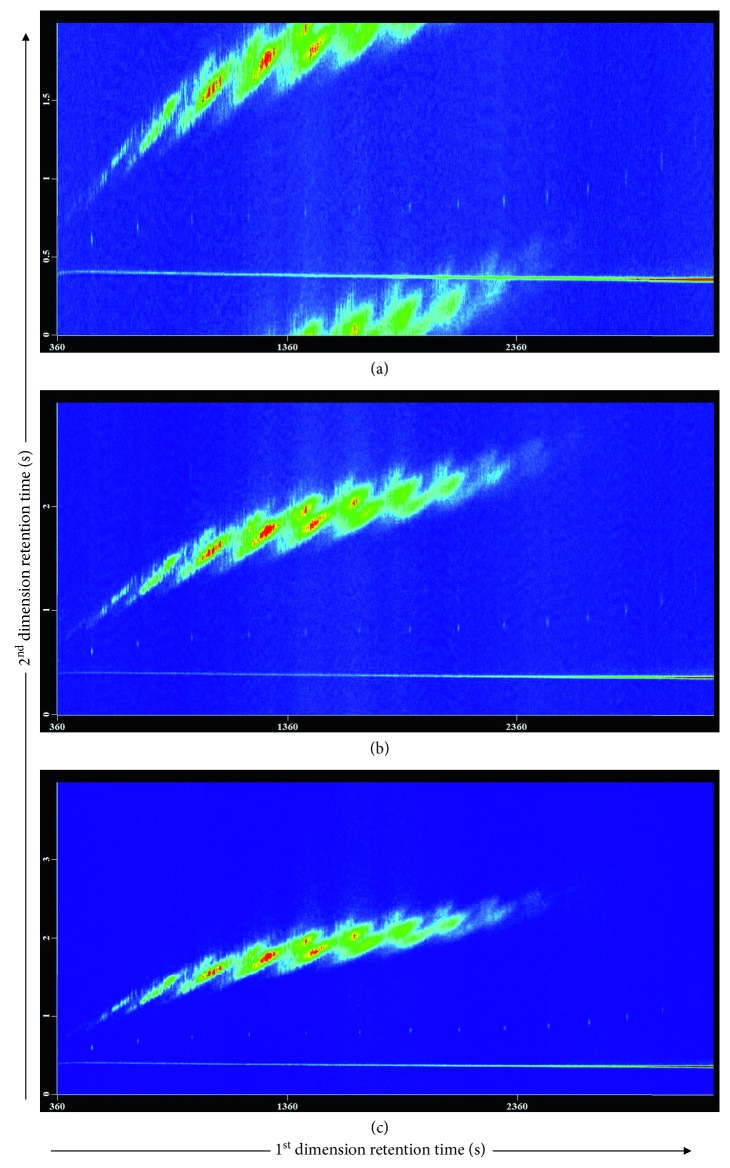
The effect of the modulation period on the separation performance of SCCP congeners: modulation period = (a) 2 s, (b) 3 s, and (c) 4 s with 20% hot pulse duration.

The modulation period, an another key factor in the GC × GC system, needs to preserve the 1D separation and elute all the compounds from two columns with the good peak shape; therefore, a relatively short modulation period is usually preferable. In this experiment, three different modulation periods (2, 3, and 4 s) with 20% hot pulse duration were investigated. At a modulation period of 2 s (Figure 3), most peaks had good shapes, but wraparound was observed for a few less-volatile SCCPs, suggesting that the current modulation period was too short to ensure the injection of all the congeners into the second column. When the modulation period was extended to 3 or 4 s, all the SCCP congeners flew out in one modulation cycle; a modulation period of 3 s (Figure 3) provided better separation and peak shapes than that obtained at 4 s (Figure 3), indicating most peaks with appropriate modulations at a 3 s modulation. Thus, a modulation period of 3 s and hot and cold pulses of 0.6 s and 0.9 s, respectively, were applied in this study.

Finally, the carrier gas flow rate and MS acquisition rate were investigated. Since the two GC columns of different dimensions are linked in series, the same flow rate will affect the separation in both columns differently. To obtain a suitable gas flow rate for both the dimensions, the flow rate ranging from 1.0 to 1.6 mL/min was tested. The results showed that a lower flow rate could slow down the elution of the sample components and improve the separation of neighboring SCCP congener groups. Moreover, a lower flow rate required a relatively low head pressure, even at high temperature, which was a benefit to the GC × GC system. Therefore, a flow rate of 1.0 mL/min was chosen in this study. The MS acquisition rate was based on the peak width of the target compounds. Since most SCCP congeners were extremely narrow of only approximately 0.1-0.2 s wide at the base, corresponding to the acquisition rate of 50–100 Hz, 100 Hz was therefore selected as the MS scan rate in this study.

**Figure 4:**
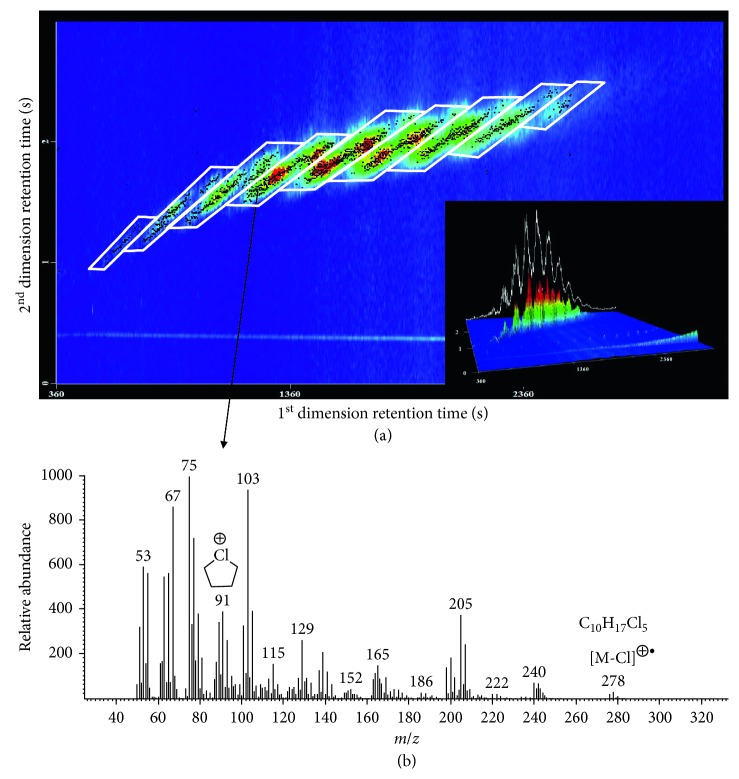
(a) GC × GC-TOF-MS chromatogram and elution pattern of a mixed SCCPs standard solution (C_10–13_, 55.5% chlorine) obtained on Rxi-5Sil MS × Rxi-17Sil MS column combination. Lower right: 3D contour plot. (b) Mass spectrum of a selected SCCP congener C_10_H_17_Cl_5_, where the fragment ion *m*/*z* 91 is the quantitative ion.

Under the abovementioned optimized conditions, the separation of SCCP congener groups was achieved. According to the distribution rules of SCCPs [24–26], the SCCPs elution patterns are identified into 9 parallel peak groups (polygons) on the 2D contour plot (Figure 4), where the congeners in each polygon have similar physicochemical properties, retention times, and the same total number of carbon and chlorine atoms. This classification not only makes the identification of SCCPs simple and intuitive but also facilitates the quantification, as the polygon areas also represent the quantitative area for SCCPs. Figure 4 shows a mass spectrum of a SCCP congener C_10_H_17_Cl_5_, where the major fragment ions at *m*/*z* 67, 75, 91, and 103 have good relative abundance while the abundance of the characteristic ion [M-Cl]^+^ (*m*/*z* 278) is very low. Although the fragment ion at *m*/*z* 91 was not the most abundant ion, this pentacyclic positive ion [C_4_H_8_Cl]^+^ was considered specific as it was only generated by the rearrangement of polychlorinated *n*-alkanes during the mass fracture process, and thus, it was selected as the quantitative ion. The other fragment ions at *m*/*z* 67, 75, and 103, although they have higher abundance than [C_4_H_8_Cl]^+^, can also be produced by other chlorinated compounds, so they were not selected as quantitative ions.

### 3.2. Fiber Selection and Optimization of HS-SPME Procedure

Initially, three different SPME fibers, including one nonpolar (100 *µ*m PDMS), one semipolar (65 *µ*m PDMS/DBV), and one highly polar (85 *µ*m PA) fibers, were investigated in terms of extraction capacity and reproducibility. Here, the extraction capacity was assessed by the peak area ratio, which was calculated by summing all the peak areas of SCCPs and dividing by the internal standard peak area; the reproducibility of the fibers was evaluated by the RSD values of peak area ratios. Each experiment was performed in triplicate with 10 mL blank water spiked at 10.0 *µ*g/L with standard mixture of SCCPs (C_10–13_, 55.5% chlorination in acetone).

As can be seen from Table 1, 100 *µ*m PDMS fiber not only provided the highest sensitivity to SCCPs but also offered the best reproducibility, whereas PA and PDMS/DBV fibers both showed lower sensitivity and poorer repeatability. These observations were basically consistent with the previous study [10, 13, 17]; therefore, 100 *µ*m PDMS fiber was chosen as the most appropriate fiber for SCCPs and used in the subsequent experiments.

**Figure 5:**
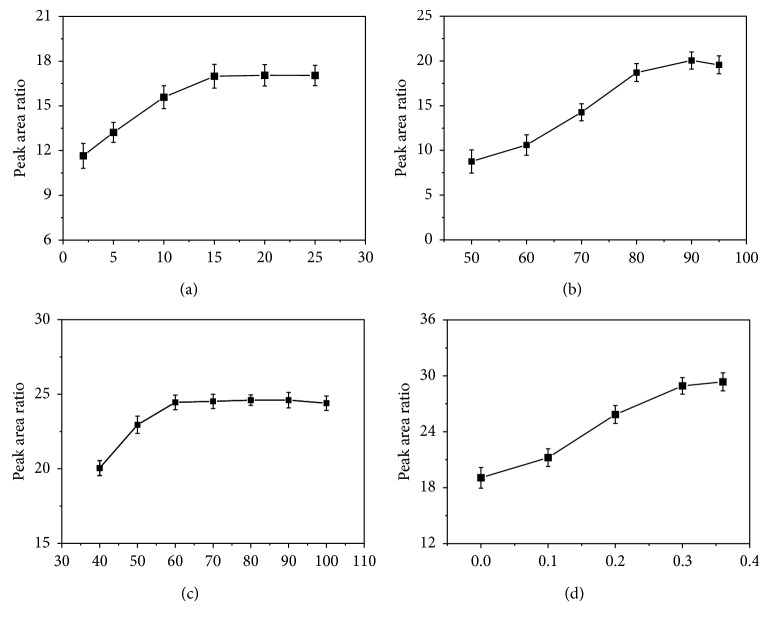
Influence of (a) incubation time, (b) extraction temperature, (c) extraction time, and (d) salt concentration on the HS-SPME extraction efficiency of SCCPs by a 100 *µ*m PDMS fiber.

After the selection of the SPME fiber, four key parameters of the extraction step were then optimized [13, 14, 17]. First, the incubation times were evaluated between 2 and 25 min.  Figure 5(a) shows that that increasing incubation time leads to an increase in SCCPs extraction from 2 to 15 min, but when the incubation time exceeded 15 min, the extraction efficiency was slightly affected. Besides, considering a shorter extraction time would enhance the experimental throughput, and 15 min was chosen as the ideal extraction time.

Extraction temperature plays an important role in the HS-SPME step but in two opposite ways: increasing temperature not only increases the analytes content in the gas phase but also accelerates the desorption of adsorbates on the fiber at the same time. In this experiment, extraction temperature was studied at 50, 60, 70, 80, 90, and 95°C with a fixed extraction time of 80 min. As can be seen from Figure 5(b), the extraction efficiency first increased with increasing temperature and reached maximum at 90°C and then dropped at 95°C. This is probably because volatilization was the main controlling factor from 50 to 90°C, while desorption became dominant when temperature reached above 90°C. Given the above, 90°C was chosen as the optimum extraction temperature.

Extraction time was further evaluated from 40 to 100 min with an interval of 10 min. In Figure 5(c), the chromatographic response showed a fast increase from 40 to 60 min and then remained almost unchanged afterwards, which means the SCCPs reached an extraction equilibrium from 60 min. Continuous increasing extraction time did not improve the extraction efficiency but reduces the experimental throughput and increases matrix interference [13], and thus, 60 min was chosen as the optimum extraction time.

Ionic strength, i.e., salt concentration, is also important for the analyte extraction in HS-SPME. Increasing ionic strength usually enhances the extraction efficiency of hydrophobic compounds through salting out phenomenon [13]. In this experiment, quantities of NaCl were added to reach 0, 0.1, 0.2, 0.3, and 0.36 (saturation concentration at room temperature) g/mL NaCl solutions. As shown in Figure 5(d), increasing salt concentration increases extraction and the best values are those obtained at the saturated concentration (i.e., 0.36 g/mL). Therefore, 0.36 g/mL of NaCl was chosen. It is worth noting that this result is a supplement to the result of Gandolfi et al. [17], who did not observe any obvious salting-out effect in the salt concentration range between 0 and 0.035 g/mL. However, in a wider salinity ranges, the salting-out phenomenon was observed and had a positive effect on the extraction efficiency.

In summary, the best HS-SPME extraction conditions are as follows: 10 mL water sample with the addition of 3.6 g NaCl was stabilized at 90°C for 15 min, then extracted for 60 min at the same temperature, and finally desorbed at 260°C for 2 min. The overall extraction time for each sample was approximately 80 min, which is less time-consuming compared to the traditional methods using LLE (120 min) [9] or SPE (130 min) [10].

### 3.3. Influence of MCCPs and LCCPs

Since MCCPs and LCCPs may also be present in the sample of SCCPs and their presence may interfere with SCCPs analysis, it is necessary to study their influence on SCCPs. To study their effect, 10 mL Milli-Q water spiked with 10 *µ*g/L mixed CPs standard solutions (C_10–13_, 55.5% chlorination; C_14–17_, 52% chlorination; and C_18–20_, 49% chlorination in acetone) were submitted to the analysis under the optimized conditions.

**Figure 6:**
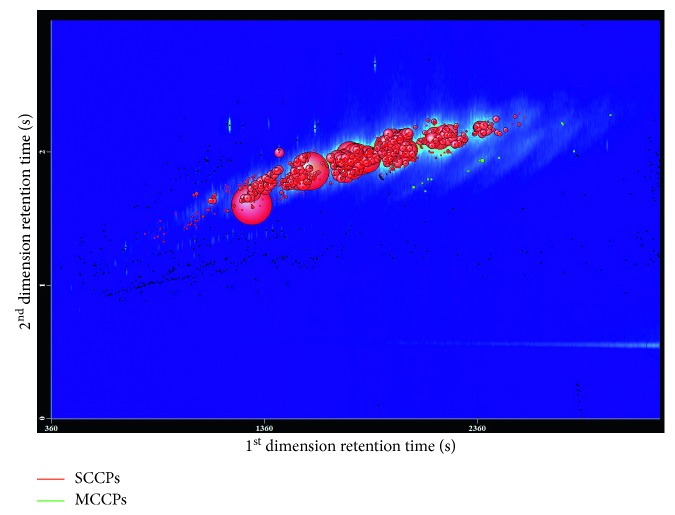
Influence of MCCP and LCCPs on the analysis of SCCPs. GC × GC-TOF-MS bubble chart obtained from a 10 *µ*g/L mixed CPs standard solution (C_10–13_, 55.5% chlorine; C_14–17_, 52% chlorine; C_18–20_, 49% chlorine) under the optimized conditions.

As can be seen from Figure 6, in the sample containing three kinds of CPs, only SCCPs show obvious signals, while MCCPs merely have weak signals and LCCPs have no signals. Although the three types of CPs had the same concentration, MCCPs and LCCPs were hardly detected under the experimental condition. This probably because SPME only extracts volatile or semivolatile components but cannot extract the less volatile components—that is, MCCPs and LCCPs cannot be enriched on the SPME fiber, so they would not interfere with the extraction of SCCPs in the initial extraction step.

Moreover, the samples were compared with those containing only SCCPs (10 *µ*g/L, C_10–13_, 55.5% chlorination in acetone). Results showed that the total peak areas of SCCPs in two kinds of samples were basically same, and the RSD values of SCCPs peak areas were less than 0.9%, indicating that the presence of MCCPs and LCCPs would not interfere with the subsequent quantification of SCCPs.

### 3.4. Method Validation

The quality parameters of the proposed method were then validated with respect to the linearity, sensitivity, accuracy, and precision to Milli-Q water, lake water, and river water samples. Calibration curves were elaborated using matrix blanks over the range of 0.2–20 *µ*g/L. The water samples used as matrix blanks were also collected from the same lake and river, merely from which SCCPs were not detected by the proposed method.  Table 2 summarizes the results.

Matrix-matched calibration curves exhibited good linearity within the calibration range, and all the correlation coefficients *R*^2^ were greater than 0.995. It is worth noting that the linearity range of this method is relatively narrow, mainly because the enrichment ability of the SPME fiber is limited. The strong enrichment ability of the SPME fiber not only pulls down the lowest point of the linearity range but also takes down the highest point of the linearity range.

The LODs and LOQs ranged from 0.06 to 0.13 *µ*g/L and 0.18–0.40 *µ*g/L, respectively. The obtained LODs were better than the one reported when using DI-SPME with GC-NCI-MS (LOD = 0.1 *µ*g/L for Milli-Q water) [10, 14], but worse than the one obtained from HS-SPME with GC-ECNI-MS (LOD = 0.004 for Milli-Q water) [17], mainly because of less sensitivity of EI-MS than ENCI-MS. Fortunately, the quality standards of SCCPs in most environmental regulations are at the level of *µ*g/L, such as 2.4 *µ*g/L for water in the Canadian Environmental Protection Act [4] and 0.4 *µ*g/L (annual average quality standard) for surface water in European Water Framework Directive [5], so the sensitivity of our method is sufficient to fulfil these criteria for the detection of SCCPs at low levels in water samples.

The accuracy of the method was evaluated by recovery experiments using the spiked sample at three levels (1, 5, and 10 *µ*g/L) in five replicates. The recoveries ranged from 82.5% to 104.2% in three types of water samples, demonstrating the good accuracy of the method.

The method precision, expressed as the RSDs of the peak areas of SCCPs at 10 *µ*g/L, was also satisfactory, with the RSD values less than 7.2% and 15% for the intra- and interday experiments, respectively.

### 3.5. Analysis of Real Samples

To confirm the applicability of this method, the proposed method was applied to ten real water samples, including two underground water samples, four lake water samples, and four river water samples. All samples were measured in duplicate.  Table 3 summarizes the average concentration of total SCCPs in the investigated water samples, and the concentration are shown as the mean ± SD. SCCPs were not detected in two groundwater water samples but detected in the lake samples (0.91–1.52 *µ*g/L) and river samples (2.98–5.07 *µ*g/L). The concentration of SCCPs in the detected river and lake samples (#3–#10) was generally greater than the values reported in the freshwater of Britain [30], Spain [14], Japan [31], and Canada [32]. Two lake samples (#3-#4) and four river samples (#7–#10) exceeded the maximum acceptable concentration quality standards of European Water Framework Directive (1.4 *µ*g/L) [5], probably because the sampling site was in the urban areas and the stock of SCCPs in China water environment was heavier than in these countries [1, 2, 33]. Considering that the mobility of SCCPs may threaten groundwater, even the trace amount of SCCPs in the water environment should be given more attention.

## 4. Conclusions

The present study is the first report which propose a green, sensitive, and reliable method for the determination of SCCPs in water samples by online HS-SPME and GC × GC-TOF-MS. Compared to the existing analytical methods, this method not only simplifies the operation process saving both time and labor but also yields good accuracy, precision, and sensitivity. The LODs (0.06–0.13 *µ*g/L) and LOQs (0.18–0.40 *µ*g/L) were relatively low and could meet the requirement of quality detection on SCCPs in water samples as mentioned in most international conventions and regulations. Besides, the interference from MCCPs and LCCPs can be excluded in the initial extraction step because these compounds cannot be enriched on the SPME fiber. Based on these observations, we believe that this method can be used for the routine analysis of SCCPs in water samples.

## Figures and Tables

**Table 1:** Extraction efficiency and reproducibility of three studied SPME fibers for extracting SCCPs.

Fiber type	Peak area ratio				RSD
	Test 1	Test 2	Test 3	Average value	(%, *n*=3)
100 *µ*m PDMS	22.39	24.16	21.45	22.66	6.06
65 *µ*m PDMS/DBV	17.43	15.63	17.62	16.89	6.51
85 *µ*m PA	17.46	16.45	13.94	15.29	8.30

**Table 2:** Analytical features of the proposed HS-SPME GC × GC-TOF-MS method for determination of SCCPs in water samples.

Water type	Linear range (*µ*g/L)	*R* ^2^	LOD (*µ*g/L)	LOQ (*µ*g/L)	Recovery (%, *n*=5)			Precision (RSD%, *n*=5)	
					1 *µ*g/L	5 *µ*g/L	10 *µ*g/L	Intraday	Interday
Milli-Q water	0.2–20.0	0.999	0.06	0.18	90.1	97.3	104	3.6	6.7
Lake water	0.2–20.0	0.996	0.10	0.31	85.4	95.8	92.6	5.8	12
River water	0.2–20.0	0.995	0.13	0.40	82.5	84.9	88.5	7.2	15

**Table 3:** Occurrence and concentration levels of SCCPs in different water samples.

Sample code	Water type	Concentration (*µ*g/L) ± SD
#1	Groundwater	n.d.
#2	Groundwater	n.d.
#3	Lake water	1.52 ± 0.08
#4	Lake water	1.40 ± 0.05
#5	Lake water	0.91 ± 0.03
#6	Lake water	1.05 ± 0.03
#7	River water	4.1 ± 0.2
#8	River water	2.98 ± 0.08
#9	River water	5.1 ± 0.1
#10	River water	3.36 ± 0.04

n.d., not detected (below the method detection limit).

## Data Availability

The data used to support the findings of this study are available from the corresponding author upon request.

## References

[1] Bayen S., Obbard O. J. P., Thomas G. O. (2006). Chlorinated paraffins: a review of analysis and environmental occurrence. *Environment International*.

[2] van Mourik L. M., Leonards P. E. G., Gaus C., de Boer J. (2015). Recent developments in capabilities for analysing chlorinated paraffins in environmental matrices: a review. *Chemosphere*.

[3] van Mourik L. M., Gaus C., Leonards P. E. G., de Boer J. (2016). Chlorinated paraffins in the environment: a review on their production, fate, levels and trends between 2010 and 2015. *Chemosphere*.

[4] Canadian Environmental Protection Act, 1999 Federal Environmental Quality Guidelines Chlorinated Alkanes, May 2018, http://www.ec.gc.ca/ese-ees/C4148C43-C35E-44EA-87A7-866E5907C42C/FEQG_Chlorinated%20Alkanes_EN.pdf

[5] Directive 2000/60/EC of the European Parliament and of the Council Establishing a Framework for the Community Action in the Field of Water Policy

[6] UNEP/POPS/POPRC.12/11/Add.3 (2016). *United Nations Environmental Programme*.

[7] UNEP/FAO/RC/COP.8/12/Add.1 (2017). *Rotterdam Convention on the Prior Informed Consent Procedure for Certain Hazardous Chemicals and Pesticides*.

[8] Zeng L. X., Li H. J., Wang T. (2013). Behavior, fate, and mass loading of short chain chlorinated paraffins in an advanced municipal sewage treatment plant. *Environmental Science and Technology*.

[9] Geiß S., Einax J. W., Scott S. P. (2010). Determination of the sum of short chain polychlorinated *n*-alkanes with a chlorine content of between 49 and 67% in water by GC-ECNI-MS and quantification by multiple linear regression. *CLEAN—Soil, Air, Water*.

[10] Castells P., Santos F. J., Galceran M. T. (2004). Solid phase extraction versus solid phase microextraction for the determination of chlorinated paraffins in water using gas chromatography–negative chemical ionisation mass spectrometry. *Journal of Chromatography of A*.

[11] Coelhan M. (2010). Levels of chlorinated paraffins in water. *CLEAN—Soil, Air, Water*.

[12] Ma X. D., Chen C., Zhang H. J. (2014). Congener-specific distribution and bioaccumulation of short-chain chlorinated paraffins in sediments and bivalves of the Bohai Sea, China. *Marine Pollution Bulletin*.

[13] Płotka-Wasylka J., Szczepańska N., de la Guardia M., Namieśnik J. (2015). Miniaturized solid phase extraction techniques. *TrAC-Trends in Analytical Chemistry*.

[14] Castells P., Santos F. J., Galceran M. T. (2003). Solid phase microextraction for the analysis of short-chain chlorinated paraffins in water samples. *Journal of Chromatography A*.

[15] Passeport E., Guenne A., Culhaoglu T. (2010). Design of experiments and detailed uncertainty analysis to develop and validate a solid phase microextraction/gas chromatography-mass spectrometry method for the simultaneous analysis of 16 pesticides in water. *Journal of Chromatography A*.

[16] Yuan S. F., Liu Z. H., Lian H. X. (2017). Simultaneous determination of eleven estrogenic and odorous chloro- and bromo-phenolic compounds in surface water through an automated online HS SPME followed by on-fiber derivatization coupled with GC-MS. *Analytical Methods*.

[17] Gandolfi F., Malleret L., Sergent M., Doumenq P. (2015). Parameters optimization using experimental design for HS solid phase micro-extraction analysis of short-chain chlorinated paraffins in waters under the European water framework directive. *Journal of Chromatography of A*.

[18] Eljarrat E., Barcelo D. (2006). Quantitative analysis of polychlorinated *n*-alkanes in environmental samples. *TrAC-Trends in Analytical Chemistry*.

[19] Tomy G. T., Stern G. A., Muir D. C. G. (1997). Quantifying C10-C13 polychloroalkanes in environmental samples by high-resolution gas chromatography electron capture negative ion high-resolution mass spectrometry. *Analytical Chemistry*.

[20] Zencak Z., Borgen A., Reth M., Oehme M. (2005). Evaluation of four mass spectrometric methods for the gas chromatographic analysis of polychlorinated *n*-alkanes. *Journal of Chromatography A*.

[21] Reth M., Zencak Z., Oehme M. (2005). New quantification procedure for the analysis of chlorinated paraffins using electron capture negative ionization mass spectrometry. *Journal of Chromatography A*.

[22] Yuan B., Wang Y. W., Fu J. J., Zhang Q. H., Jiang G. B. (2010). An analytical method for chlorinated paraffins and their determination in soil samples. *Chinese Science Bulletin*.

[23] Gao W., Wu J., Wang Y. W., Jiang G. B. (2016). Quantification of short- and medium-chain chlorinated paraffins in environmental samples by gas chromatography quadrupole time-of-flight mass spectrometry. *Journal of Chromatography A*.

[24] Korytár P., Parera J., Leonards P. E. G. (2005). Characterization of polychlorinated *n*-alkanes using comprehensive two-dimensional gas chromatography-electron-capture negative ionisation time-of-flight mass spectrometry. *Journal of Chromatography A*.

[25] Xia D., Gao L., Zhu S., Zheng M. H. (2014). Separation and screening of short-chain chlorinated paraffins in environmental samples using comprehensive two-dimensional gas chromatography with micro electron capture detection. *Analytical and Bioanalytical Chemistry*.

[26] Xia D., Gao L. R., Zheng M. H. (2016). A novel method for profiling and quantifying short- and medium-chain chlorinated paraffins in environmental samples using comprehensive two-dimensional gas chromatography–electron capture negative ionization high-resolution time-of-flight mass spectrometry. *Environmental Science and Technology*.

[27] Reth M., Oehme M. (2004). Limitations of low resolution mass spectrometry in the electron capture negative ionization mode for the analysis of short- and medium-chain chlorinated paraffins. *Analytical and Bioanalytical Chemistry*.

[28] Moore S., Vromet L., Rondeau B. (2004). Comparison of metastable atom bombardment and electron capture negative ionization for the analysis of polychloroalkanes. *Chemosphere*.

[29] Gao Y., Zhang H. J., Zou L. L. (2016). Quantification of short-chain chlorinated paraffins by deuterodechlorination combined with gas chromatography-mass spectrometry. *Environmental Science and Technology*.

[30] Nicholls C., Allchin C., Law R. (2001). Levels of short and medium chain length polychlorinated *n*-alkanes in environmental samples from selected industrial areas in England and Wales. *Environmental Pollution*.

[31] Iino F., Takasuga T., Senthikumar K., Nakamura N., Nakanishi J. (2005). Risk assessment of short-chain chlorinated paraffins in Japan based on the first market basket study and species sensitivity distributions. *Environmental Science and Technology*.

[32] Houde M., Muir D. C. G., Tomy G. T. (2008). Bioaccumulation and trophic magnification of short- and medium-chain chlorinated paraffins in food webs from Lake Ontario and Lake Michigan. *Environmental Science and Technology*.

[33] Jiang W. Y. H., Huang T., Mao X. X. (2017). Gridded emission inventory of short-chain chlorinated paraffins and its validation in China. *Environmental Pollution*.

